# Diagnostic Yield in Childhood-Onset Hearing Loss: A Meta-Analysis and Systematic Review

**DOI:** 10.3390/life16040610

**Published:** 2026-04-07

**Authors:** Shahar Taiber, Ryan J. Carlson, Nidal Muhanna, Rani Abu Eta

**Affiliations:** 1 Department of Otolaryngology/Head, Neck and Maxillofacial Surgery, Tel Aviv Sourasky Medical Center, Tel Aviv 6423906, Israel; 2 Gray Faculty of Health Sciences, Tel Aviv University, Tel Aviv 6997801, Israel; 3 Departments of Genome Sciences and Medicine, University of Washington, Seattle, WA 98195, USA

**Keywords:** hearing loss, deafness, exome sequencing, WES, genetic diagnosis

## Abstract

This systematic review and meta-analysis aims to determine the diagnostic yield of whole-exome and targeted-panel sequencing in children with hearing loss. We searched PubMed, Google Scholar, and the Cochrane Library to identify studies describing cohorts of >50 families undergoing whole exome or targeted panel sequencing. Studies were excluded if they pre-screened for common deafness genes without including the data in final analyses, focused on syndromic hearing loss, or lacked diagnostic yield information. Meta-analysis employed a random-effects model of single proportions to determine yield across included studies. The pooled diagnostic yield for bilateral hearing loss was ~47%, while unilateral cases demonstrated a yield of only ~5% across both testing methods. These findings demonstrate that the diagnostic yield for bilateral hearing loss exceeds that of other conditions frequently requiring clinical genetic testing, such as epilepsy and intellectual disability, though this advantage does not extend to unilateral hearing loss. These results have important implications for healthcare policy decisions regarding genetic testing guidelines and funding.

## 1. Introduction

Hearing loss (HL) is the most prevalent sensory deficit in humans, affecting approximately 1 in 1000 newborns [1,2]. Detection typically occurs through two primary pathways—routine newborn screening programs where available, or clinical investigation prompted by delayed language development [3]. The causes of HL are diverse, and include in utero infections, ototoxic medication exposure, hyperbilirubinemia, and genetic mutations, with genetic factors estimated to account for 50% of all cases [1,4].

Over 150 genes have been implicated in nonsyndromic hearing loss (NSHL) over the last several decades, and many more have been linked to syndromes that include hearing impairment [5], which has driven the widespread adoption of next generation sequencing (NGS) as a tool for genetic diagnosis. A genetic diagnosis carries significant implications for patients and families, informing family planning decisions and guiding clinical management of affected patients. This is particularly crucial in cases of syndromic HL initially presenting as nonsyndromic, where early genetic identification can prompt essential clinical evaluations and preventive treatments [2,6,7]. The importance of genetic diagnosis will continue to increase with the emergence of gene therapies, as evidenced by current clinical trials for OTOF-related deafness and ongoing preclinical development for other genetic targets [8,9].

In recent years, genetic workup has typically utilized NGS in the form of targeted panel sequencing (TPS) with comprehensive panels targeting all genes known to be involved in HL, sometimes preceded by an initial screen for prevalent mutations such as those found in GJB2 or SLC26A4 [10,11]. As the number of known HL genes has increased, the size of TPS panels has grown proportionally. The gold standard sequencing panel from the University of Iowa, OtoSCOPE (v9), now includes 244 genes for syndromic and nonsyndromic HL [12]. With sequencing costs ever shrinking, many studies have now adopted whole exome sequencing (WES) as the diagnostic tool of choice, with downstream genomic analysis typically focusing primarily on the known HL genes as above. In published cohort sequencing studies of pediatric HL, the diagnostic yield of NGS varies considerably, ranging from 13% to 73% [13,14]. This variation is largely influenced by factors specific to each cohort, such as consanguinity rates, family history of HL, severity of HL, and ethnic background [1,15,16]. While the impact of these factors on diagnostic success is well-established, a systematic analysis of diagnostic rates provides essential evidence to inform clinical guidelines and healthcare funding decisions.

Despite clear clinical benefits to genetic diagnosis in many cases of childhood-onset HL, healthcare coverage for NGS testing is not uniform. Several countries with primarily public healthcare coverage have incorporated these NGS tests, but coverage varies widely in nations with private insurance coverage [4,17,18]. Therefore, providing clear data on the expected diagnostic yield of NGS can guide policy decisions and communication with insurance companies. These data may also be useful in the prioritization of NGS vs. other diagnostic modalities, i.e., imaging, as is performed in other conditions such as epilepsy and retinal dystrophies [19,20]. The emergence of effective gene therapies can also increase the clinical utility of genetic testing and accelerate its coverage in healthcare systems. For example, the remarkable success of gene therapy for spinal muscular atrophy has led multiple countries to fund genetic testing for this condition [21,22]. Similar dynamics may apply to HL as targeted therapies emerge, highlighting the importance of understanding diagnostic yields in this population.

## 2. Materials and Methods

### 2.1. Search Strategy and Sources

Relevant studies were identified by PubMed, Google Scholar, and Cochrane Library queries. The search was restricted to studies published in English between 1 January 2010, and 10 October 2024. These dates were chosen because widespread use of WES started in 2010 and therefore NGS studies on HL were rarely found before this period. The following search terms were used: inherited hearing loss, hereditary hearing loss, genetic deafness, genetic etiology, genome sequencing, exome sequencing, WES, panel, genetics of hearing loss, genetic diagnosis, childhood, children, hearing loss, deafness, cochlear implant, genetic testing, nonsyndromic hearing loss, targeted sequencing, yield, cohort. Table S1 contains all the search terms and search-term combinations.

Next, a 2-step filtration process was employed. After removal of duplicates, titles and abstracts were screened to exclude reviews, case-reports, non-original articles, books, and other studies which clearly did not meet the inclusion criteria. Next, studies were reviewed in-depth to further narrow the publication list based on availability of the sequencing results within the text and for cohort characteristics as described below. We adhered to the Preferred Reporting Items for Systematic Reviews and Meta-Analyses (or PRISMA) guidelines [23]. All included studies were analyzed by ST and RJC and any disagreements between were resolved through discussion and mutual consensus.

### 2.2. Selection Process

Because small cohort are more likely to be biased, studies were included only if the cohort size was >50 families with childhood-onset HL and if genetic diagnosis was completed by NGS (either WES or TPS). We did not exclude studies based on severity of HL, family history, consanguinity rates, or ethnicity.

Studies were excluded if participating families were pre-screened for common deafness genes, such as GJB2, and then excluded from downstream analyses of diagnostic yield (as this would distort the calculated diagnostic yield), if studies focused primarily on syndromic HL, or if studies did not include information on diagnostic yield. Table S2 contains all the studies that passed the initial title and abstract screen along with the exclusion reason for those that were omitted from further analysis.

### 2.3. Data Extraction

Clinical characteristics and relevant data from the included studies were tabulated, including the year of publication, age of onset, presence of syndromic features, laterality, exclusion of genes, sequencing method, size of cohort, country of origin, total number of families tested, and the number of individuals receiving a positive genetic diagnosis. Of note, some studies were not entirely constrained to sensorineural HL but also included some cases of conductive or mixed HL. However, these represented very small fractions of the patients, and therefore the data were treated as primarily representing sensorineural HL in all analyses. In addition, not all studies explicitly reported the laterality of hearing loss. When laterality was reported, bilateral hearing loss data were used for Figure 2 and unilateral hearing loss data for Figure 3. When laterality was not specified, data were included only in Figure S2 or Figure S3 according to sequencing method. Diagnostic yield was defined as the proportion of individuals receiving a pathogenic or likely pathogenic (P/LP) variant diagnosis according to ACMG/AMP classification criteria. Variants of uncertain significance (VUS) were explicitly excluded from all yield calculations. Heterozygous variants in autosomal recessive genes were not counted as positive findings unless a second pathogenic variant was identified in trans, consistent with established criteria for biallelic diagnosis.

### 2.4. Statistical Analysis and Data Presentation

Meta-analyses were performed using a random-effects model in R version 4 (R Core Team, Vienna, Austria) using the metaphor 4.6 package. Pooled proportions were estimated using the Freeman–Tukey double arcsine transformation [24]. Heterogeneity was quantified using the I^2^ statistic and τ^2^, and is reported for each subgroup analysis. Risk of bias across included studies was assessed using the Joanna Briggs Institute (JBI) checklist for prevalence studies (Table S3). Publication bias was assessed using the Doi plot and LFK index (Figure S1).

## 3. Results

### 3.1. Study Selection

**Figure 1:**
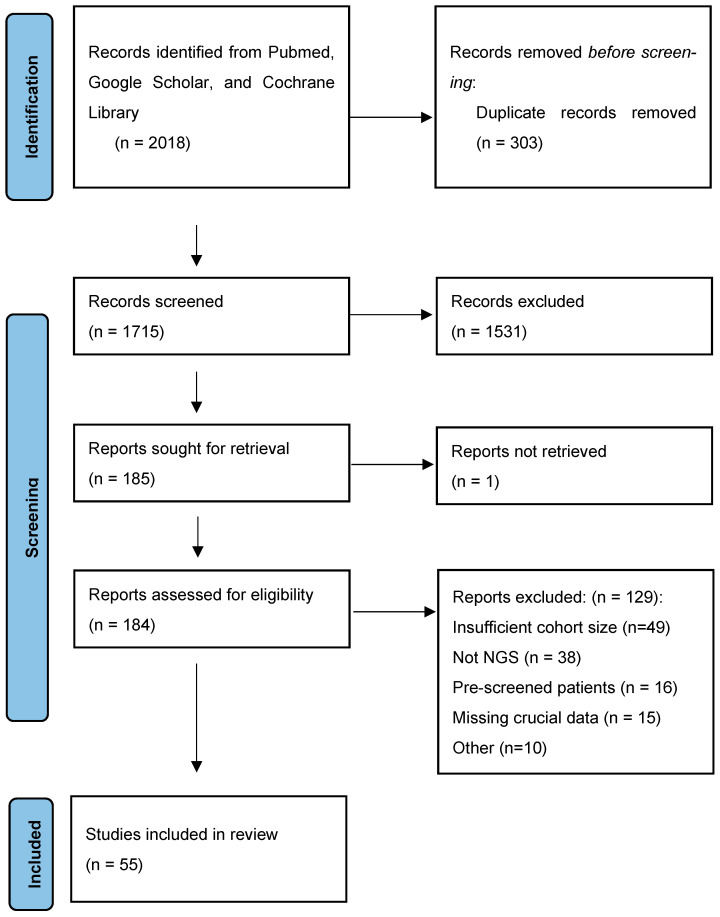
Flowchart of search strategy and filtration.

Of the 2018 records identified from PubMed, Google Scholar, and Cochrane Library, 303 duplicates were removed, leaving 1715 records for title and abstract screening. Following exclusion of 1531 records, 185 studies were sought for retrieval, of which 1 could not be retrieved. Of the 184 reports assessed for eligibility, 129 were excluded. A total of 55 studies were included in the systematic review (Figure 1). Risk of bias assessment using the JBI checklist for prevalence studies revealed that no studies were classified as high risk; the majority were rated low risk, and four were rated medium risk (Table S3). Publication bias was assessed using the Doi plot and LFK index; the LFK index was 0.02, indicating no meaningful asymmetry (Figure S1).

### 3.2. Results of Syntheses

**Figure 2:**
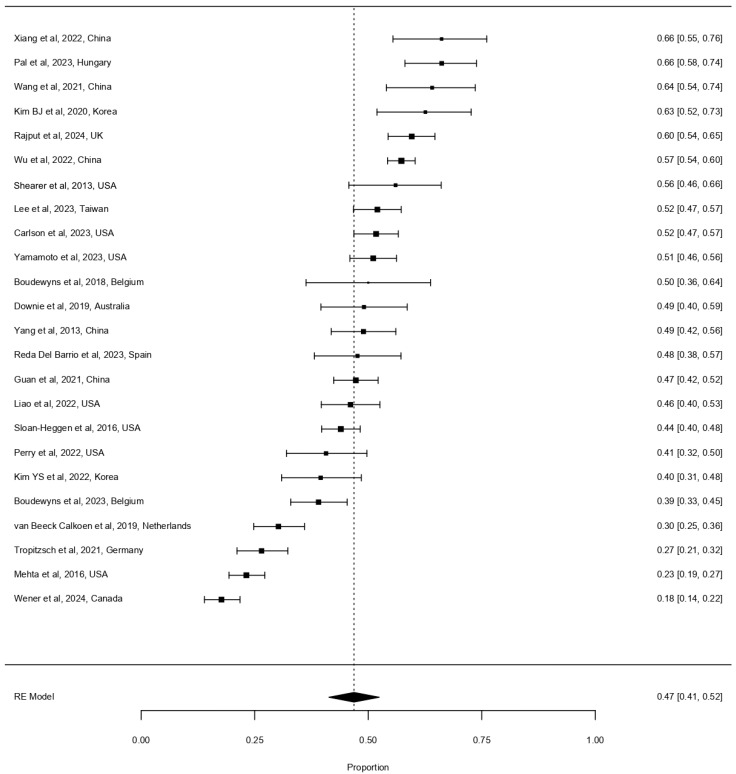
Forest plot depicting the diagnostic yield of next-generation sequencing for bilateral childhood-onset hearing loss [2,12,16,25,26,27,28,29,30,31,32,33,34,35,36,37,38,39,40,41,42,43,44,45]. Each line corresponds to a study included in the analysis. For each entry, diagnostic yield is listed with 95% confidence interval in parentheses. Square size correlates to cohort size. The diamond at the bottom of the plot shows the pooled estimated diagnostic yield. determined by the inverse-variance method under a random-effects model. The dashed vertical line represents the pooled estimate; the diamond represents the pooled effect size with its 95% confidence interval. Total number of patients included: 6399.

A random-effects meta-analysis was performed to evaluate the diagnostic yield of genetic testing across several clinical subgroups. The main analysis focused on bilateral childhood-onset HL, which comprised the majority of included studies (Figure 2). In some studies, a small fraction of syndromic or late-onset HL cases was included where no subgroup breakdown was available; these were treated as representative of the bilateral sensorineural population. The overall pooled diagnostic yield across all included studies was 46.9% (95% CI 41.3–52.4%), with high heterogeneity (I^2^ = 94.8%, τ^2^ = 0.0175).

**Figure 3:**
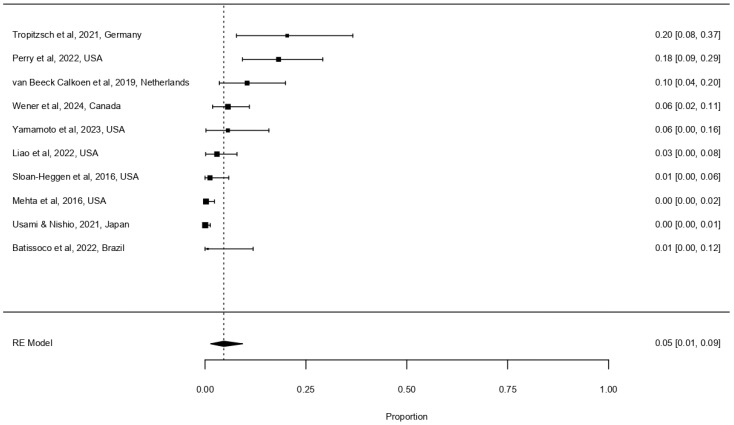
Forest plot depicting the diagnostic yield of next-generation sequencing for unilateral childhood-onset hearing loss [16,32,38,39,40,43,44,45,49,50]. Format as described for Figure 2. Total number of patients included: 842.

Given that the clinical utility of genetic testing in unilateral HL remains less well established and must be weighed against the cost of NGS [31,46,47], we also evaluated diagnostic yield in this subgroup (Figure 3). Because the pooled proportion was near zero (~5%), a generalised linear mixed model (GLMM) was used in place of the standard random-effects model, as Wald-type confidence intervals are unreliable at the distributional boundary [48]. The pooled diagnostic yield for unilateral childhood-onset HL was 4.7% (95% CI 1.4–9.3%, I^2^ = 82.8%, τ^2^ = 0.0151).

**Figure 4:**
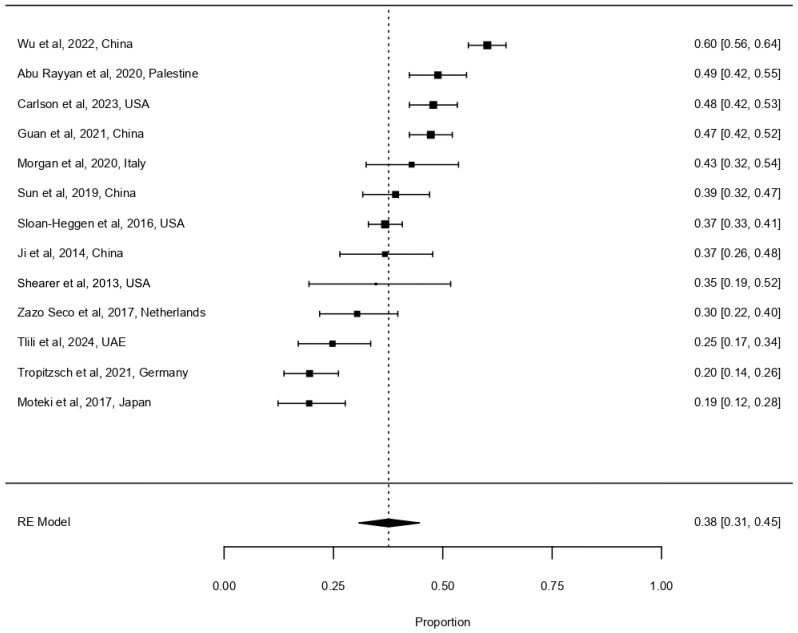
Forest plot depicting the diagnostic yield of next-generation sequencing for sporadic childhood-onset hearing loss [1,2,12,16,30,37,39,51,52,53,54,55,56]. Format as described for Figure 2. Total number of patients included: 2884.

Because some studies have suggested that a positive family history significantly increases diagnostic yield [1,16,32], we additionally analysed the subset of sporadic cases—those with no reported family history, as these represent a particularly common and clinically relevant scenario. Pooling data from both TPS and WES to maximise statistical power, the diagnostic yield in sporadic cases was 37.6% (95% CI 30.8–44.7%, I^2^ = 92.8%, τ^2^ = 0.0152, Figure 4).

To evaluate the effect of sequencing method on diagnostic yield, studies were stratified by WES and TPS regardless of laterality (Figures S2 and S3, respectively). The pooled diagnostic yield using TPS was 45.0% (95% CI 38.1–52.0%, I^2^ = 93.7%, τ^2^ = 0.0242), and using WES was 42.0% (95% CI 36.7–47.4%, I^2^ = 97.7%, τ^2^ = 0.0232). The difference in performance between the two methods was not statistically significant (*p* = 0.479).

The high heterogeneity observed across all subgroups is not unexpected, reflecting the variable clinical characteristics, patient inclusion criteria, and bioinformatic pipelines of the contributing studies. To explore potential sources of heterogeneity, we examined whether year of publication was associated with diagnostic yield. No significant correlation was found (Spearman ρ = −0.14, *p* = 0.326, Figure S4), suggesting that neither the progressive expansion of gene panels over time nor the adoption of standardised ACMG/AMP variant classification criteria following their introduction in 2015 materially influenced reported yields across this literature.

We also examined whether cohort size was associated with diagnostic yield, given that our inclusion threshold of 50 or more families was necessarily arbitrary. Spearman rank correlation between cohort size and diagnostic yield was weak and non-significant (ρ = −0.23, *p* = 0.099, Figure S5), indicating no systematic relationship between study size and reported yield. This was further supported by sensitivity analyses restricting the meta-analysis to studies with cohorts of ≥100 and ≥200 participants, which yielded pooled estimates of 41.5% (95% CI 36.7–46.4%) and 37.6% (95% CI 31.4–43.9%), respectively, both consistent with the primary analysis.

## 4. Discussion

In this meta-analysis of NGS testing methods in patients with childhood-onset HL, encompassing approximately 22,000 participants in total, we demonstrate a pooled diagnostic yield of 46.9% for bilateral childhood-onset hearing loss. This yield is comparable to those reported in inherited retinal diseases and substantially higher than those observed in other conditions [20,21].

Our analysis identified only a modest reduction in diagnostic yield for cases without family history, contrasting with previous findings [1,32]. In the included studies, family history is characterised as having another family member with childhood-onset HL, which could include parents, grandparents, or siblings. Therefore, this discrepancy may be explained by demographic characteristics of the studied populations. For example, the Palestinian population studied by Abu Rayyan et al. is characterised by high consanguinity and high fertility rates, factors that increase the likelihood of recessive inheritance patterns and the probability of identifying multiple affected family members [1]. In contrast, Far Eastern populations, which comprise 69% of our cohort, have comparably low fertility rates [57]. In these populations, the probability of identifying familial cases is therefore inherently lower, not necessarily due to genetic factors but because individuals are less likely to have siblings.

Our analysis demonstrates a striking difference in diagnostic yields between unilateral and bilateral HL approximately 4.7% versus 46.9%, respectively. For the unilateral subgroup, a GLMM was used to obtain reliable confidence intervals given the proportion’s proximity to zero. The 4% yield is lower than what has been described in studies evaluating unilateral HL specifically [31,46,58], which may reflect differences in the definition of a positive genetic finding. For example, Gruber et al. report that 50% of patients with unilateral HL had a “genetic abnormality”, including GJB2 heterozygotes who would not be regarded as solved cases by other studies [46]. In addition, some studies group all asymmetric HL patients together, while others make a distinction between asymmetric and unilateral HL [31,58]. These results correspond to a number needed to test of 2.1 individuals to identify one case of genetic bilateral HL versus 21.3 for genetic unilateral HL. This substantial disparity has important clinical implications for the often lengthy, exhausting, and costly diagnostic journey faced by families of children with HL. The markedly lower yield in unilateral cases suggests that prioritising other diagnostic modalities, such as cCMV testing and MRI, may be more efficacious in these cases [46]. Importantly, in some cases HL may present initially as unilateral before progressing to the contralateral ear, making the age at testing a critical factor in the application of these statistics in practice [59]. These findings provide evidence-based guidance for healthcare policymakers, particularly in systems where diagnostic yield thresholds determine public funding for genetic testing.

Among sporadic cases, the pooled diagnostic yield was 37.6%, only modestly lower than the overall bilateral estimate. This is clinically relevant, as sporadic presentation is a common scenario in practice, and these findings support the utility of genetic testing even in the absence of a positive family history.

The similar performance between TPS and WES, 45% and 42%, respectively, a difference that was not statistically significant, likely reflects the current practice of focusing downstream bioinformatic analysis on known HL loci, even when WES is used. Without a proven link to HL, variants in genes not typically included in a TPS panel are more likely to be classified as variants of uncertain significance (VUS), which were excluded from all yield calculations in this analysis. Notably, families with negative TPS results may have limited potential for identifying a genetic etiology through subsequent WES. However, the continuing decline in sequencing costs and the closing cost gap between panels and exome reagents suggest that WES will soon become the standard NGS approach. WES also offers the possibility of reanalysis when additional novel variants and genes are linked to HL in the future.

## 5. Limitations

This study has several limitations. First, because a comprehensive breakdown of yield based on clinical features such as severity was not typically available in the included studies, our analysis represents a relatively broad definition of childhood-onset HL. Since it has been shown that diagnostic yield increases with severity, it is possible that the true diagnostic yield for severe-profound bilateral childhood-onset HL could be as high as 60%, while for mild-moderate it could be as low as 25%, as previously suggested [16,32]. The underlying composition of participants in terms of severity within each study is therefore likely a contributor to the observed heterogeneity.

Second, formal meta-regression to systematically decompose sources of heterogeneity was not feasible, as the majority of included studies report only aggregate diagnostic yields without individual-level clinical data. We note, however, that year of publication did not significantly correlate with diagnostic yield, suggesting that neither the progressive expansion of gene panels over time nor the phased adoption of ACMG/AMP variant classification criteria following their introduction in 2015 materially influenced reported yields. Furthermore, cohort size was not significantly associated with diagnostic yield, and sensitivity analyses restricting to cohorts of ≥100 and ≥200 participants yielded similar pooled estimates, supporting the validity of our 50-family inclusion threshold.

Third, we did not evaluate the diagnostic yield for syndromic HL, as this would vary substantially between syndromes and there is a paucity of studies examining a pooled syndromic population. We acknowledge that the definition and exclusion of syndromic hearing loss may vary across studies due to differences in clinical workup protocols worldwide, and we therefore relied on the reported classification by the original investigators, excluding only studies that explicitly focused on specific syndromic conditions.

Finally, as is true for all meta-analyses combining cohorts from different settings, residual heterogeneity is expected given differences in clinical practices between centres and healthcare systems. This includes variability in hearing testing methods, the presence of newborn hearing screening programmes, access to genetic counselling and testing, bioinformatic pipelines, and criteria for variant interpretation. Risk of bias assessment using the JBI checklist for prevalence studies confirmed that no included study was rated as high risk; the predominance of moderate-risk ratings reflects the clinic-based recruitment design common across this literature rather than study-specific quality deficiencies.

## 6. Conclusions

This comprehensive systematic review establishes NGS as a high-yield diagnostic tool for bilateral childhood-onset HL, with diagnostic rates of 38–47% that exceed those of many other conditions routinely covered by healthcare systems. The stark contrast between bilateral and unilateral HL yields provides crucial evidence for clinical decision-making and resource allocation, suggesting that in unilateral cases alternative diagnostic modalities should be prioritised before NGS. These findings provide an evidence-based framework to inform healthcare policy decisions regarding genetic testing coverage and to accelerate the implementation of genetic testing for the millions of children affected by genetic HL worldwide.

## Data Availability

All data used to generate the figures in the manuscript can be found in the Supplementary Tables. Any additional data, such as codes or intermediate analyses will be made available by authors upon request.

## Supplementary Materials

The following supporting information can be downloaded at: https://www.mdpi.com/article/10.3390/life16040610/s1, Table S1: Search terms and search-term combinations; Table S2: List of studies that initially passed the title and abstract screen; Table S3: Risk of bias assessment; Table S4: Studies used to create Figure 2; Table S5: Studies used to create Figure 3; Table S6: Studies used to create Figure 4; Table S7: Studies used to create Figure S2; Table S8: Studies used to create Figure S3; Figure S1: DOI plot of included studies; Figure S2: Diagnostic yield of whole-exome sequencing for childhood-onset hearing loss; Figure S3: Diagnostic yield of targeted-panel sequencing for childhood-onset hearing loss; Figure S4: Spearman rank correlation between year of publication and diagnostic yield across included studies; Figure S5: Spearman rank correlation between cohort size and diagnostic yield across included studies.

## Abbreviations

The following abbreviations are used in this manuscript:

HL	Hearing loss
TPS	Targeted-panel sequencing
WES	Whole-exome sequencing
NGS	Next-generation sequencing
