# Supplementary material for: Diagnostic Yield in Childhood-Onset Hearing Loss: A Meta-Analysis and Systematic Review
Source: Life (Basel). 2026 Apr 7;16(4):610. doi: 10.3390/life16040610 (PMC13117509; doi:10.3390/life16040610)
Supplement: Supplementary file 1 [file life-16-00610-s001.zip › Supplementary Figures.pdf]

Supplementary Information for:

Diagnostic Yield in Childhood-Onset Hearing Loss: a Meta-Analysis and Systematic Review

Figure S1: DOI plot of included studies

Figure S2: Diagnostic yield of whole-exome sequencing for childhood-onset hearing loss

Figure S3: Diagnostic yield of targeted-panel sequencing for childhood-onset hearing loss

Figure S4: Spearman rank correlation between year of publication and diagnostic yield across included studies

Figure S5: Spearman rank correlation between cohort size and diagnostic yield across included studies

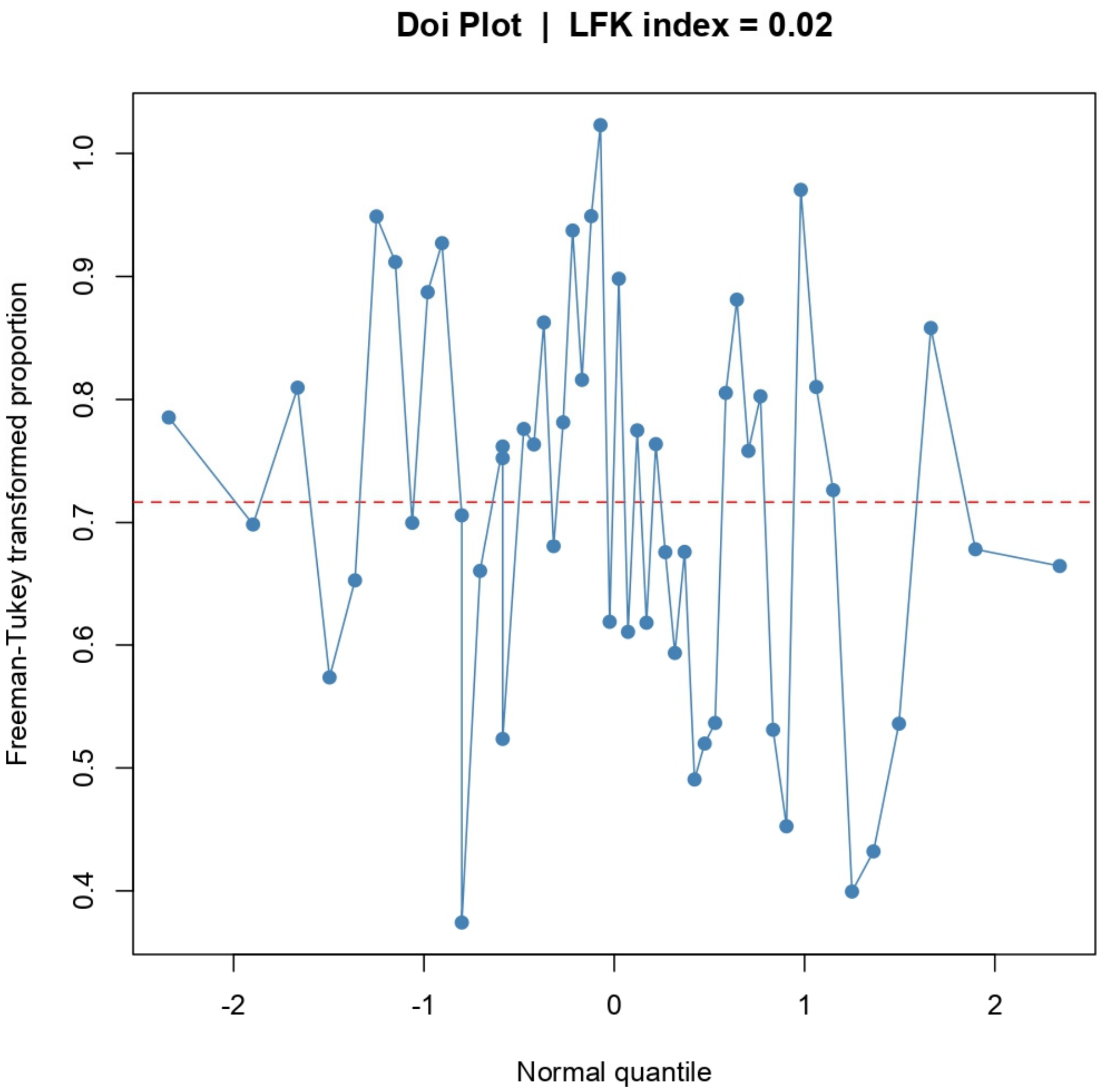

Figure S1: Doi plot for assessment of publication bias. Each circle represents one included study plotted on the Freeman–Tukey transformed scale. The LFK index was 0.02, indicating no asymmetry. The dashed horizontal line represents the pooled effect estimate on the transformed scale.

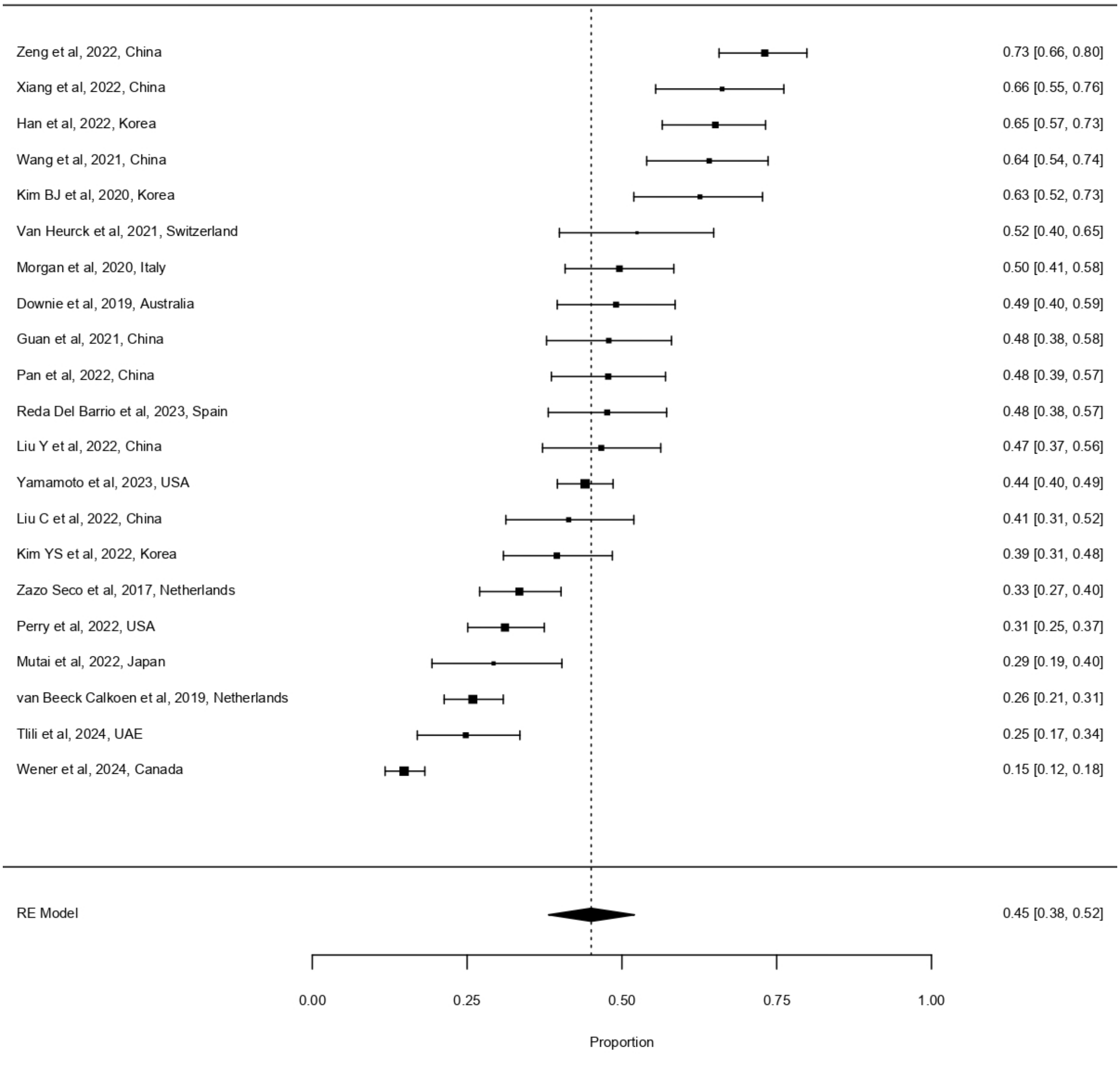

Figure S2: Forest plot depicting the diagnostic yield of whole-exome sequencing for childhood-onset hearing loss. Format as described for Figure 2. Total number of patients included: 19,734.

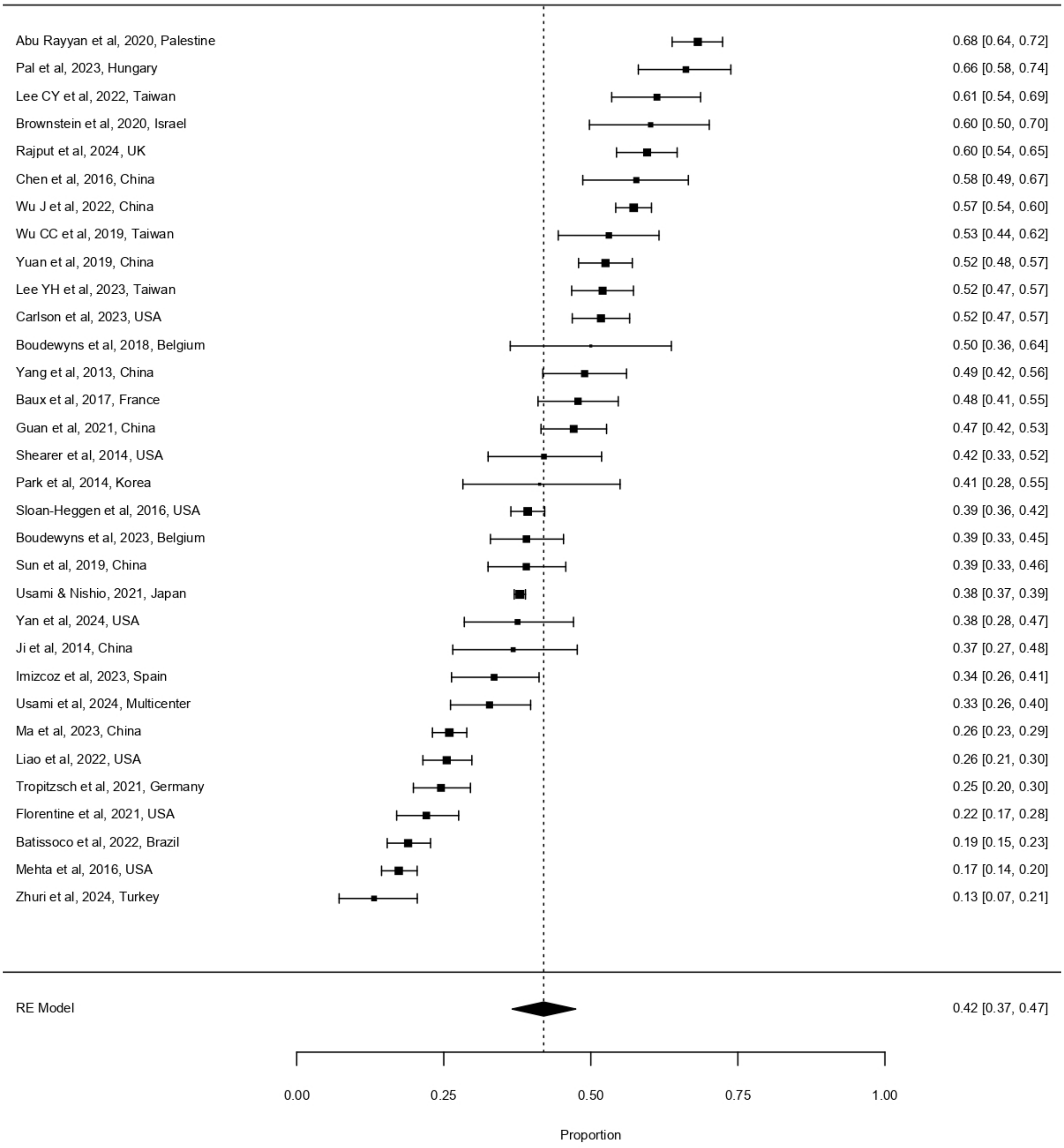

Figure S3: Forest plot depicting the diagnostic yield of targeted-panel sequencing for childhood-onset hearing loss. Format as described for Figure 2. Total number of patients included: 3,332.

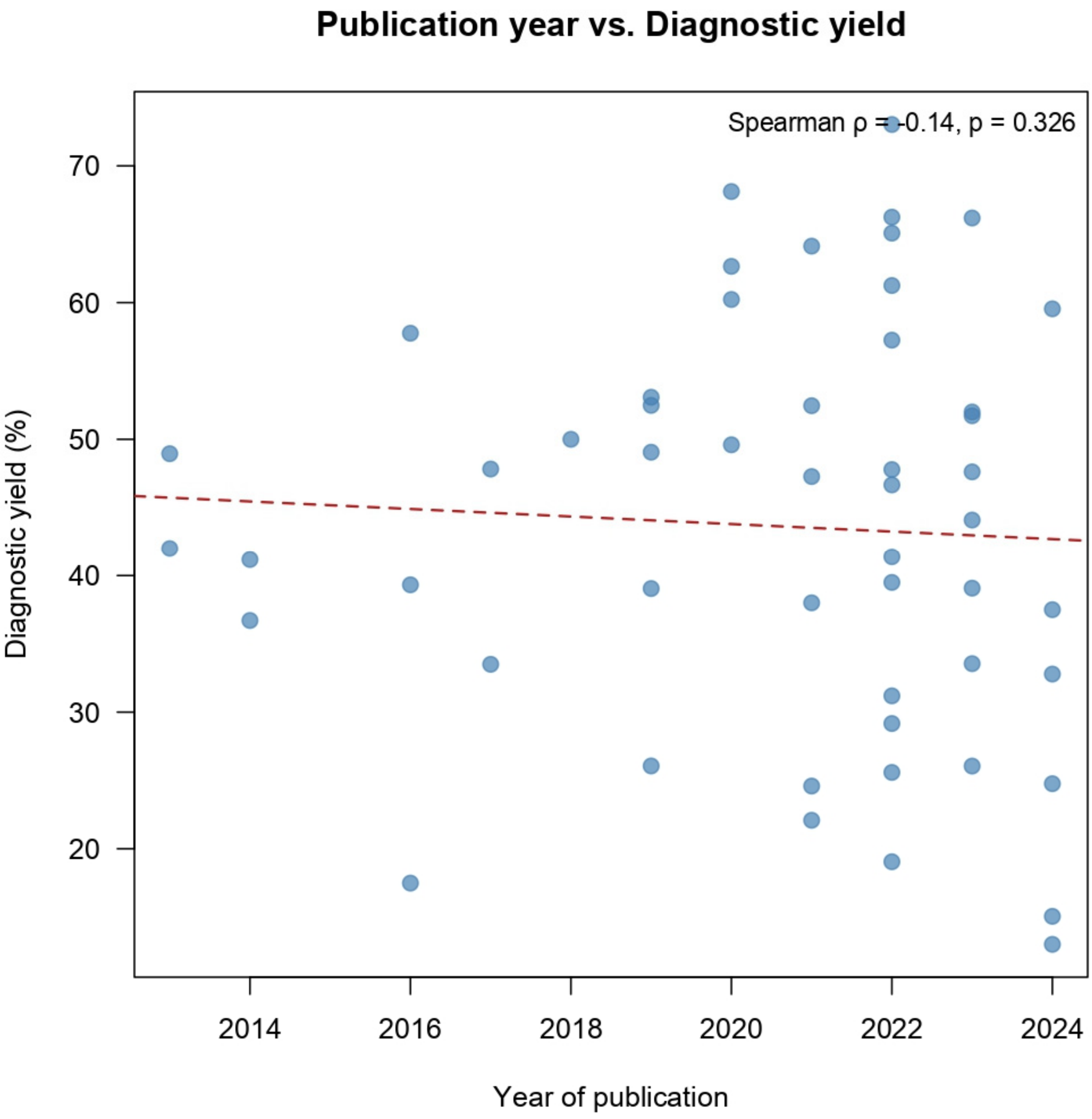

Figure S4: Spearman rank correlation between year of publication and diagnostic yield across included studies.

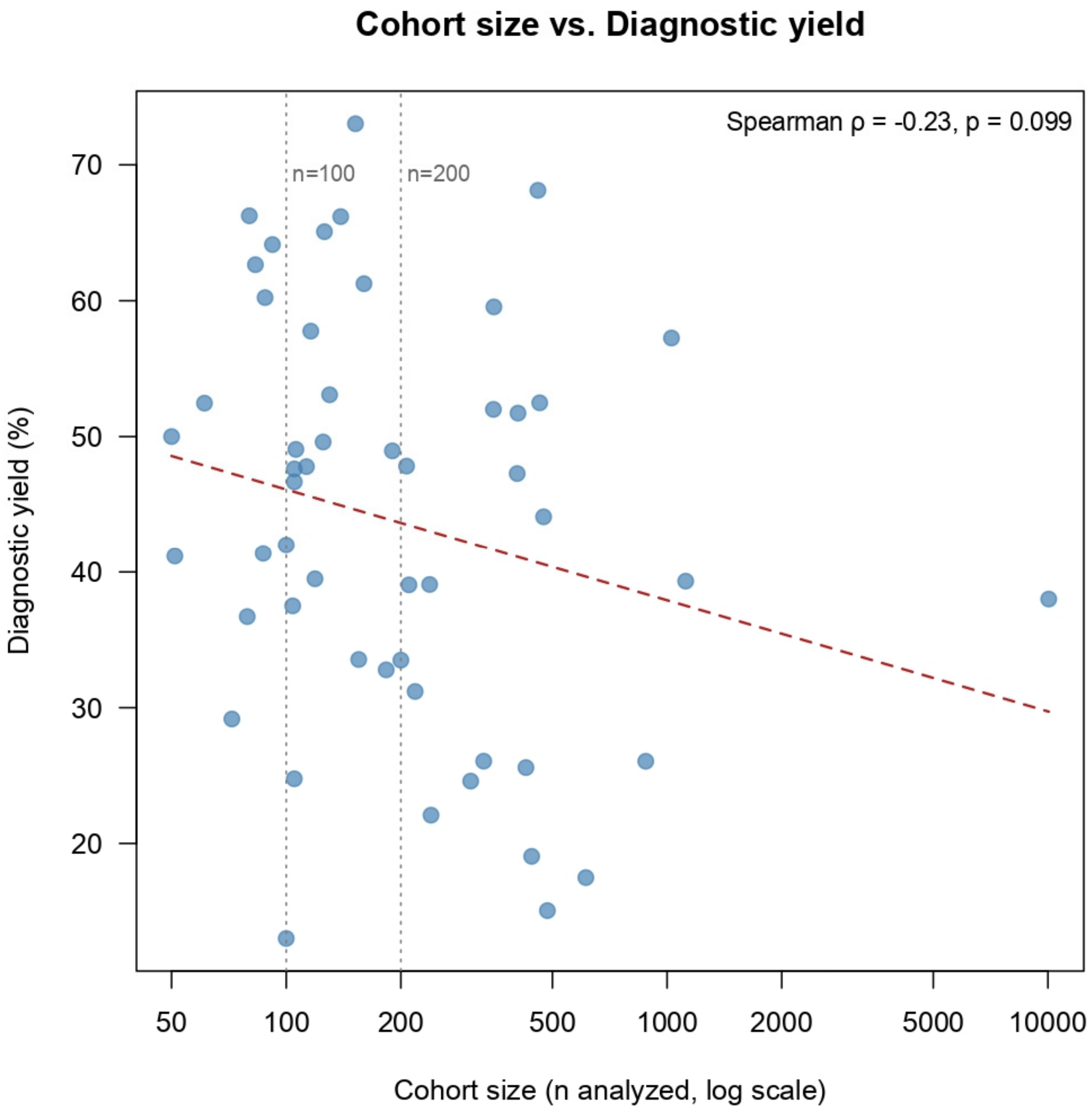

Figure S5: Spearman rank correlation between cohort size and diagnostic yield across included studies.
